# Cardiovascular Health Among Employees of a Brazilian Tertiary Hospital Assessed by the Life’s Essential 8 Score: A Cross-Sectional Pilot Study

**DOI:** 10.3390/jcm15083134

**Published:** 2026-04-20

**Authors:** Erlon Oliveira de Abreu-Silva, Fernanda Jafet El Khouri, João Gabriel Sanchez, Angela Cristine Bersch-Ferreira, Alexandre Biasi, Timo Siepmann, Aline Marcadenti

**Affiliations:** 1 Hcor Research Institute, Hospital do Coracao, Sao Paulo 04004-030, Brazil; erlon@terra.com.br (E.O.d.A.-S.); fjkhouri@hcor.com.br (F.J.E.K.); joaogsanchez@gmail.com (J.G.S.); amarcaden@hcor.com.br (A.M.); 2 Division of Health Care Sciences, Dresden International University, 01067 Dresden, Germany; 3 Beneficência Portuguesa de São Paulo, São Paulo 01323-001, Brazil; 4 Department of Neurology, Medical Faculty, University Hospital Carl Gustav Carus, TUD Dresden University of Technology, 01069 Dresden, Germany; 5 Graduate Program in Health Sciences (Cardiology), Instituto de Cardiologia/Fundação Universitária de Cardiologia do Rio Grande do Sul, Porto Alegre 90040-371, Brazil; 6 Graduate Program in Public Health, Faculdade de Saúde Pública da Universidade de São Paulo, São Paulo 01246-904, Brazil

**Keywords:** cardiovascular disease, cardiovascular health, cardiovascular risk factors, prevention

## Abstract

**Background/Objectives**: The American Heart Association Life’s Essential 8 (LE8) is a tool proposed to categorize overall cardiovascular health (CVH), ranging from 0 to 100 and classifies CVH as low (<50), moderate (50–79) or high (≥80), based on the following health behaviors (diet, physical activity, nicotine exposure and sleep) and health factors (body mass index—BMI, lipid levels, glycemic profile and blood pressure). Although used in the general population, it is not part of the health assessment routine in the workplace. We assessed CVH of healthcare workers using an LE8-based score through a mobile application. **Methods**: Cross-sectional pilot study with adults working at a tertiary hospital in Brazil. We used an app for self-reporting LE8 metrics. Additionally, data on age, sex, and mental health (10-item Perceived Stress Scale, PSS-10) were collected. **Results**: Sixty-five adults (58.5% female; mean age 36 ± 9.01 years) were included. The mean LE8 overall score was 69.39 ± 12.63. The proportion of participants in the low, moderate and high cardiovascular health categories were 6.2%, 69.2% and 24.6%, respectively. Diet quality (34.76 ± 24.3) and physical activity (45.38 ± 40.58) were in the “low cardiovascular health” category. “Health behaviors” had a significantly lower mean score than “health factors” (58.90 ± 20.53 vs. 79.88 ± 15.55, *p* < 0.001). The mean PSS-10 score was 19.01 ± 7.49, indicating moderate perceived stress. Overall LE8 and PSS-10 scores were not significantly correlated (r_s_ = −0,0.17; *p* = 0.161). There was no significant difference in the mean overall LE8 score in the linear regression model adjusting for age, sex and perceived stress. **Conclusions**: Among employees of a Brazilian tertiary hospital, the adapted LE8 score indicated overall moderate CVH. Health behaviors—particularly diet quality and physical activity—were the main vulnerable areas. Implementation of an LE8-based assessment in the workplace may be useful for targeted prevention strategies in Brazil. Future larger and longitudinal studies are warranted to confirm these findings.

## 1. Introduction

The importance of cardiovascular disease (CVD) as a matter of public health rests clearly in the analysis of data regarding the incidence, costs and morbimortality associated with this condition [1]. Additionally, the high prevalence and low rates of control of traditional and novel cardiovascular risk factors such as hypertension (HTN), type 2 diabetes mellitus (T2DM), hyperlipidemia and overweight/obesity, as well as smoking, sedentary lifestyle, poor diet quality and sleep deprivation [2,3].

For many years, healthcare was based on the stratification of cardiovascular risk according to the presence of classic risk factors. More recently, however, a novel approach was proposed, focused on the evaluation of cardiovascular health (CVH) using dedicated scores elaborated by the American Heart Association (AHA): first the Life’s Simple 7 (LS7) [4] and, currently, the Life’s Essential 8 (LE8) [5]. Both aggregate behavioral variables, such as diet, physical activity and sleep are classified as classic risk factors [4,5]. Ranging from 0 to 100, with cut-offs for low (<50), moderate (≥50 to 79) and high (≥80) CVH, (an overall higher score shows better health, with both tools, and is related to lower CVD and overall mortality [6,7].

In other countries, the evaluation of employees’ cardiovascular health, as part of an institutional health and well-being program, was associated with a reduction in absenteeism and workforce health-related costs [8,9,10], as well as decreased cardiovascular risk and mortality [11,12,13,14]. In order for the LE8 to be adequately used as this health indicator in the working environment, the AHA suggests the following points to be addressed: (1) employees must acknowledge their own health (i.e., they must know their own score); (2) the proportion of workers in each stratum of the score must be known; (3) use of longitudinal metrics related to cardiovascular health; and (4) the magnitude of the variation in each metric during time must be measured [5,15].

In Brazil, previous studies reported a mean LE8 in the moderate CVH category (63.4 ± 13.1) [16], with an estimated prevalence of low, moderate, and high adherence to the LE8 total factors of 11.8%, 74%, and 14.2% [17]. The use of such scores to evaluate the workforce health among healthcare providers is not frequent, even in hospitals or other healthcare institutions, including those specialized in cardiology. To the best of our knowledge, there is no published data about this topic in this specific setting. Accordingly, this pilot study aimed to evaluate the cardiovascular health, based on the LE8, using an app for cell phones and/or tablets, among employees of a tertiary hospital. The findings of the present study will guide the implementation of the LE8 score on a larger scale as part of the institution’s workforce healthcare program.

## 2. Materials and Methods

### 2.1. Study Design

Cross-sectional study with employees from Hcor—Associação Beneficiente Síria from whom data were collected using the institution’s department of occupational medicine—Cuidar Hcor—app for cell phones and tablets.

### 2.2. Eligibility Criteria

Adult males and females who worked in any sector of the hospital and had given informed consent, via app, were eligible to participate in the study.

### 2.3. Cuidar Hcor App and the In-App CVH Assessment Score

The Cuidar Hcor app has been available, free of charge, for employees since before this study. The development of the in-app score was a partnership between different departments of our institution: Research Institute, Cuidar Hcor, Information Technology, and Inovação Hcor.

During the period of study, the in-app score was advertised on the hospital’s intranet webpage, and the use was stimulated by the attending physicians from Cuidar Hcor to those who attended medical appointments. The potential benefits and instructions on how to use and fill in the information in the LE8 score were available at the “more info” section of the in-app functionality, similar to other electronic apps. Subjects could use the in-app score and choose not to participate in the study by not giving consent.

### 2.4. The LE8 Score

The score used in this research was based on the LE8, which evaluates the degree of cardiovascular health from the analysis of eight risk factors that can be improved by adopting a healthier lifestyle: smoking/nicotine exposure, physical activity, body mass, eating habits, blood pressure (BP), serum glucose/hemoglobin A1c (Hb_A1c_) and cholesterol, and duration of sleep. These metrics are divided into two main areas: health behaviors (diet, physical activity, nicotine exposure and sleep) and health factors (body mass index—BMI, lipid levels, glycemic profile and blood pressure) [5]. Details about the indicators, cut-offs/metrics and the respective scores for the evaluation of each one of these domains are in the Supplementary Material (Table S1).

According to AHA recommendations, some metrics on diet quality assessment were adapted to reflect Brazil’s local reality. Hence, we used the Cardiovascular Health Diet Index (CHDI) to assess the quality of diet. This is a system of points, ranging from 0 to 110, developed to evaluate how much a diet is aligned with cardiovascular disease prevention [18]. CHDI was specifically adapted to the Brazilian diet culture from AHA guidelines [19], and evaluates 11 components: fruits, vegetables, whole grains, nuts/seeds, legumes, dairy products, sweetened beverages, red meat, processed meat and ultra-processed food. Higher scores reflect a better quality of diet. Details on the specifics of CHDI can be found in the Supplementary Material.

According to AHA, LE8 results must be presented as a continuous score from 0 to 100, obtained through the unweighted average of the values for each one of the eight evaluated elements [5]. The use of a graphical depiction of the whole cardiovascular health spectrum is encouraged and, in some cases, so is the use of a categorized approach to general cardiovascular health where low, moderate and high levels are determined by scores of 0 to 49, 50 to 79 and 80 to 100, respectively [5].

### 2.5. Data Acquisition

Data were self-reported, since information was collected using the Cuidar Hcor app 1.0. Information on the definitions, metrics and cut-offs of the LE8 parameters, as well as for the diet questionnaire, was also available for participants as a “?” icon beside the box for entering the requested data. Additionally, subjects were instructed to use their more recent laboratory exam results to fill in the related information on blood levels of glucose/hemoglobin A1c (Hb_A1c_) and blood lipids, as well as their more recent measured values of BP, body weight and height.

### 2.6. The PSS-10

Besides the variables from the LE8, information about age, sex and self-perception of stress through the 10-item Perceived Stress Scale (PSS-10) was collected. This scale asks about feelings and thoughts during the previous month [20] (Supplementary Material, Box S1), and responders must answer on a 5-level Likert scale: 0 (never), 1 (almost never), 2 (sometimes), 3 (almost always) and 4 (always). To calculate the result, values from questions 4, 5, 7 and 8 must be inverted (0 = 4, 1 = 3, 2 = 2, 3 = 1, 4 = 0) and then, all answers are summed up. Values range from 0 to 40 and categories of low, moderate and high self-perceived stress are defined by scores of <14, 14 to 26 and >26, respectively [20].

### 2.7. Outcomes

The primary outcome of our study was the overall cardiovascular health score obtained using the adapted LE8, in points. The secondary outcome was the characterization of the health status based on each individual metric of the LE8, considering the prevalence of overweight/obesity, HTN, T2DM, hyperlipidemia and smoking, as well as emotional health, quality of diet and time of sleep and physical activity.

### 2.8. Statistical Analysis

Since it was a pilot study, we used a convenience sample with no formal sample size calculation. Data were summarized in absolute and relative frequencies and measures of central tendency (mean, median) and variability (standard deviation, interquartile range) according to the type and distribution of the variables.

Exploratory analyses were performed using the Wilcoxon signed rank test to compare the rank sum of the overall LE8 score, as well as the LE8 scores related to the health factors and health behaviors, and also the PSS-10 score between men and women; also we analyzed the correlation between total LE8 scores and PSS-10 scores using the Spearman correlation coefficient and evaluated a linear regression model where the overall LE8 score was the outcome and age, sex, and PSS-10 score were the predictors. Given the final small sample size, and the exploratory nature of the analyses, we opted for using non-parametric tests. However, the authors present the mean of the adapted LE8 and PSS-10 scores, rather than the median, given the better interpretability of the former. Entries with missing data were excluded from our analysis.

All statistical analyses were performed on a two-tailed α of 0.05. All analyses were conducted with the Stata 19.0 BE software (Statacorp, College Station, TX, USA).

### 2.9. Ethical Considerations

The study is in accordance with the principles outlined in the Declaration of Helsinki and the regulations and requirements from the CEP/CONEP system (National Commission of Ethics in Research) and was approved by the local ethics committee (Comitê de Ética em Pesquisa do Hcor; Approval Code: CAAE 68991423.6.0000.0060; Approval Date: 10 May 2023). All participants gave their written informed consent before inclusion in the study. The study’s protocol was registered at Plataforma Brasil (CAAE: 68991423.6.0000.0060).

## 3. Results

A total of 78 individuals filled out the adapted LE8 score, via app, between May 2023 and June 2024. Entries with incomplete data (13) were not considered for analysis.

From the 65 participants with complete data, 38 (58.5%) were female and the mean age was 36 ± 9.01 years. Table 1 summarizes the baseline characteristics of our sample. The routine use of glucose-, blood pressure- and lipid-lowering medication was reported by 2, 9 and 5 subjects, respectively.

**Figure 1:**
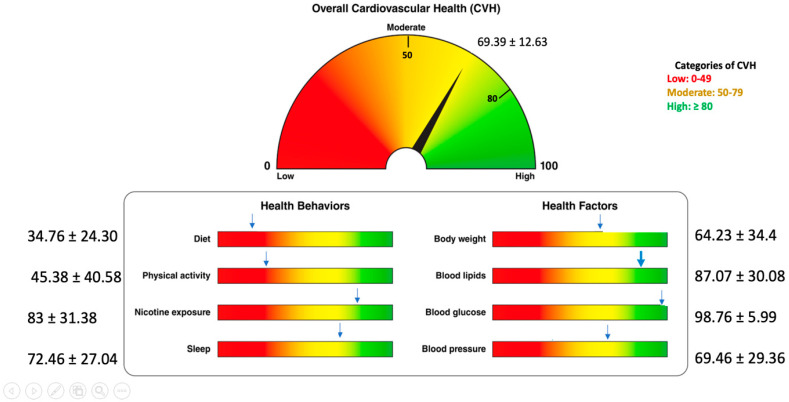
Average score on LE8 (overall and for each individual parameter). Adapted from Lloyd-Jones DM et al. [5].

Figure 1 depicts the primary outcome. In our sample, the average adapted LE8 overall score was 69.39 ± 12.63. The proportion of participants in the low, moderate and high cardiovascular health categories were 6.2%, 69.2% and 24.6%, respectively. The mean score for the “health factors”, 79.88 ± 15.55 points, was higher than the one for the “health behaviors”, 58.90 ± 20.53 points, and the sum of ranks associated with the health factors is significantly higher than the one associated with the health behaviors (*p* < 0.001). The findings on the quality of diet evaluation are summarized in Table 2.

The mean score on PSS-10 was 19.01 ± 7.49, at the moderate self-perceived stress category. Table 3 summarizes the comparisons of scores for the overall LE8, health factors, health behaviors and PSS-10 between men and women. There was no significant correlation between the overall LE8 and the PSS-10 scores (r_s_ = −0.17; *p* = 0.161). After adjusting for age, sex and PSS-10 score, there was no significant difference in the mean overall LE8 in the multivariable linear regression model (Table 4).

## 4. Discussion

In this pilot study of employees from a Brazilian tertiary hospital, four main findings emerged. First, assessment of cardiovascular health using an adapted, app-based Life’s Essential 8 score was feasible in the workplace setting. Second, overall cardiovascular health was in the moderate range, with significantly lower scores for health behaviors compared with health factors. Third, diet quality and physical activity were the weakest components, despite a relatively low prevalence of traditional clinical risk factors. Finally, overweight/obesity was independently associated with lower overall LE8 scores, whereas perceived stress was not significantly correlated with cardiovascular health in this sample.

The evaluation of the cardiovascular health of employees from a tertiary hospital in Brazil using a self-administered LE8-based score, via app, showed mean score values compatible with the moderate cardiovascular health stratum, with lower scores for “health behaviors” compared to “health factors”. Additionally, self-perception of stress among participants was moderate.

The importance of such an evaluation, using dedicated and validated tools, grows alongside the emphasis on the need for a change in the paradigm from a care based on the treatment of the disease, to one centered on prevention and health to optimize resources with, among other things, the direct and indirect reduction in costs. In this sense, the use of such tools may be viewed as just the beginning, since the body of evidence shows that the most effective model is the one combining the utilization of one of these scores—as a screening tool—with a structured care program [10,21,22].

A sub-analysis of a cohort of the Framingham Heart Study offspring shows that the overall score on LE8 was inversely associated with the risk of CVD [6]. Data from the NHANES (National Health and Nutrition Examination Survey) study show that having higher scores on LE8 was associated with lower total and CVD-related mortality [7], while evidence from the UK Biobank replicates these findings and, in addition, shows that LE8 is capable of detecting a significant increase in the risk of incident CVD in a median follow-up of 10.3 years in individuals whose LE8 score varied from the moderate to the low cardiovascular health stratum [23].

There is also evidence that the LE8 is capable of detecting subclinical atherosclerotic CVD [24,25,26]. Liu et al. had shown that LE8 scores at the high cardiovascular health stratum are related to a significantly lower risk of abdominal aortic calcification [24]. Analyses in Swedish adults aged 50 to 64 years showed that LE8 had an inverse and dose–response relationship with the presence of carotid atherosclerotic plaques among those with scores compatible with low cardiovascular health [23], and that these same individuals presented a significantly higher risk of having positive coronary artery calcium scores [26].

Within the paucity of data on the use of such scores in the Brazilian population, it is worthy to mention two sub-analyses from the ELSA-Brasil (Brazilian Longitudinal Study of Adult Health) study showing that high scores on the LE8 were associated with lower cognitive decline in an 8-year follow-up [25] and with lower frequency and severity of migraine [26]. In these studies, the mean LE8 score was 63.4 ± 13.1 [16], with 74% of participants allocated to the moderate CVH stratum, while 11.8% and 14.2% were in the low and high CVH categories, respectively [17].

These findings highlight the importance of initiatives to promote, at the population level, higher scores across time, considering their association with relevant clinical outcomes beyond the cardiovascular system.

In this scenario, health assessment at the workplace becomes an interesting opportunity since the so-called biometric screening—measurement of physical characteristics and aerobic fitness that can be performed at the workplace and used in the periodic workforce health evaluation—can be combined with a risk assessment tool and, together, be the starting point of an institutional health and well-being program.

The use of such a strategy at the workplace has been shown to be effective in the early detection of cardiovascular risk factors and control of established conditions aiming at the prevention of future events [11,12,13,14]. The Kailuan study, in China, enrolled employees from a factory and showed that an institutional program of hypertension control and treatment was related to lower blood pressure levels and, also, lower total mortality rates [11]. The assessment of cardiovascular health by LE8 in that population showed that higher scores were related to more years free of CVD [12] and, also, to lower risk of subclinical atherosclerosis [13]. Additionally, it was demonstrated that the risk of stroke was reduced or reversed among those who improved their score over time [14].

In our sample, health assessment using an adapted LE8-based score was able to detect, beyond the estimate of cardiovascular health, the low prevalence of traditional risk factors such as HTN, T2DM, smoking and hyperlipidemia, as well as the low adherence to current recommendations on diet and engagement in physical activity. In addition, the tool also detected a prevalence of overweight/obesity compatible with the national mean [27].

These findings of lower scores for the “health behaviors” driven by the findings on diet and physical activity, as well as the high proportion of persons with excess weight, agree with an NHANES sub-analysis [28]. The study showed that, between 2005 and 2018, scores related to nicotine exposure, blood lipids and sleep time had improved, while those related to glycemia and BMI got worse and there was no significant change in the scores related to blood pressure, diet and physical activity. Noteworthy is that the latter two scores remained at the low cardiovascular health stratum for the whole follow-up. Consequently, there was no significant change in the overall LE8 score between 2005 and 2018 [28].

Another important point is the inverse and robust association between LE8 scores and the self-perception of health as well as mental and physical health-related quality of life. Investigators for the SCAPIS (Swedish Cardiopulmonary Bioimage Study) study have shown that self-perception of poor health and low physical and mental health-related quality of life were significantly more frequent in subjects scoring 40 compared to those scoring 80 on LE8, after adjusting for age, sex and geographical region [29]. Differently, in our sample, the correlation between the adapted LE8 and PSS-10 was weak and not significant, although negative.

It is important to acknowledge that people who work in healthcare, from administrative workers to physicians [30,31,32], have been shown to be subjected to a work environment that poses important barriers to a healthy lifestyle [33] that translates into an increased risk of CVD, some known cardiovascular risk factors (such as T2DM and obesity), musculoskeletal injuries and cancer [34,35,36,37], but also stress, physical inactivity and sleep deprivation [35,36,38,39]. These findings underscore the fact that the work in a hospital is dynamic and stimulating but, also, involves unhealthy, arduous and difficult activities from both physical and psychological standpoints, especially when the routine of inflexible schedules, long work shifts and, in some cases, multiple jobs is considered [40]. Even though these features are common to all healthcare-related roles, there may be specific variations within and between professions. This does not change the fact that, ultimately, healthcare professionals are ordinary people with similar health habits compared to those of the general population, despite the training and theoretical knowledge on health-related issues [41].

Interestingly, in our study, in opposition to previous publications [6,7,11,12,13,14,22,23,24,25,26], there was no significant difference in the mean scores between men and women.

Our study has limitations. It was a pilot study in which the sample was small and presented baseline characteristics that can be different from the general adult population, since the mean age was low, limiting generalizability. This can preclude the study from making any robust inference on why any association may or may not have been found as well as the reason for any difference or similarity with results from previous studies. Additionally, final sample size and characteristics, as well as completeness and possibility of verification of data accuracy may have been affected by the sole availability of the adapted score for self-report via app, since there was no systematic utilization of this tool in the routine clinical setting and the use of virtual platforms is still more frequent among the young. Recall, measurement and desirability bias cannot be ruled out. Another point to be highlighted is that we had no information regarding the specific job or hospital sector in which the participants work in the hospital. Arguably, results may differ according to the specific occupation within the healthcare field, although the literature shows a pattern fitting the healthcare workers as a group. In addition, by the inherent nature of the study, statistical inferences are all exploratory and results shall be interpreted accordingly.

Our study has strengths. In our country there is scarce information on the current status of cardiovascular health, both in and out of the workplace. Our project shows that an initiative like the use of a dedicated score for such assessment is feasible and capable of giving important information regarding points to be approached, and their priority levels, when an institutional health and well-being program is to be implemented. Although exploratory, our analysis highlights the importance of excess weight as a prevalent risk factor to health and the need for structured and long-term multiprofessional strategies for its management, with emphasis on the adoption of healthier diet habits and an increase in time dedicated to physical activity, even in a younger population. We adapted our diet questionnaire to one that has already been validated and used in the Brazilian population. This allowed us to properly evaluate this domain of the LE8 with a minimized risk of under- or overestimating the quality of our participants’ diet because we considered the particularities of our people’s eating habits. Similar adaptations to assess diet quality in accordance with a country or region’s eating habits have been previously published [42,43,44]. This adaptation increases the validity of our findings and their comparability to other studies that used LE8 and followed the same methodological refinement on this topic, as well as those using the original LE8 in its entirety, since CHDI was validated from the AHA diet guidelines [18]. In addition, we assessed the subjects’ mental health status using a simple and validated tool and showed that the self-perception of stress among persons working in a tertiary hospital is considerable and must be addressed more thoroughly. As a matter of fact, the authors of the scores proposed by AHA, studying the nature of the metrics used and strategies for improvement, acknowledge that psychological health and well-being are paramount, but subjacent to all metrics, and, therefore, opted not to include them as specific parameters of LS7 [4] and LE8 [5]. Its evaluation is encouraged for determining the level of global health status, nevertheless [5].

## 5. Conclusions

In this pilot study, cardiovascular health among employees of a Brazilian tertiary hospital, assessed using an app-based Life’s Essential 8 score, was in the moderate range. While traditional clinical risk factors were relatively well controlled, health behaviors—particularly diet quality and physical activity—represented the main areas of vulnerability. The implementation of a digital LE8-based assessment in the workplace may serve as a practical screening tool to guide targeted prevention strategies. Future larger and longitudinal studies, using LE8 both as a digital app and as part of the traditional daily clinical routine, are warranted to confirm these findings and to evaluate whether structured institutional health programs can sustainably improve cardiovascular health metrics over time in Brazil.

## Figures and Tables

**Table 1:** Baseline clinical characteristics. SD, standard deviation; mmHg, millimeters of mercury; mg/dL, milligrams per deciliter; IQR, interquartile range.

	N = 65
Age, years, mean (SD)	36 (±9.01)
Female sex, *n* (%)	38 (58.5%)
Body mass index, median, (IQR)	27.8 [23.2; 31.0]
Blood pressure, mmHg, mean (SD)	113.6/72.6 (±8.6/10.5)
Total Cholesterol, mg/dL, mean (SD)	188.2 (±60.7)
Fasting glucose, mg/dL, mean (SD)	87.1 (±8.7)
Hemoglobin A1c, %, mean (SD)	5.1 (±0.7)
Physical activity, minutes/week, mean (SD)	90.1 (±130.3)
Sleep, hours/night, mean (SD)	6.3 (±1.4)
Smoking (previous, current or living with a person who smokes), *n* (%)	26 (39.9%)

**Table 2:** Quality of diet.

**Foods**	**Intake**	
Fruits	1.69 ± 1.08 portion/day	
Vegetables	1.49 ± 1.06 portion/day	
Grains	1.4 ± 1.53 portion/day	
Fish	0.59 ± 0.72 portion/week	
**Beverages**	**Prevalence**	**Intake**
Soft drinks	69.2%	2.24 ± 1.33 glasses/week
Industrialized juice	62.3%	2.75 ± 3.78 glasses/week
Chocolate milk	46.1%	3.6 ± 11.81 glasses/week
**Routine use**		
Industrialized frozen food	43%	
Meals in restaurants	50.7%	

**Table 3:** Comparison of mean LE8 (overall, for health factors and for health behaviors) and PSS-10 scores between men and women (Wilcoxon rank sum test).

	Men	Women	*p*
Overall LE8 score	68.40 ± 2.53	70.09 ± 2.00	0.892
LE8 score for Health Factors	77.31 ± 3.13	81.71 ± 2.42	0.240
LE8 score for Health Behaviors	59.49 ± 3.81	58.48 ± 3.44	0.0774
PSS-10 score	17.25 ± 1.41	20.26 ± 1.20	0.111

**Table 4:** Multivariable linear regression model evaluating the mean overall LE8 adjusting for sex, age and PSS-10 score. LE8—Life’s Essential 8; CI: confidence interval; * continuous; PSS-10: 10-item Perceived Stress Scale.

	Variation on Overall LE8	95% CI	*p*
Age *	0.22 (/year)	−0.32 to 0.37	0.896
Sex	−2.80	−9.27 to 3.67	0.390
PSS-10 score *	−0.37	−0.80 to 0.59	0.089

## Data Availability

Data may be made available upon direct contact with the first author (eoabreu@hcor.com.br) pending evaluation of the request according to the publication policy of Hcor Research Institute.

## Supplementary Materials

The following supporting information can be downloaded at: https://www.mdpi.com/article/10.3390/jcm15083134/s1, Table S1. Life’s Essential 8 detailed metrics; Table S2. Quality of diet—Details of the assessment in the study; Box S1. 10-item Perceived Stress Scale (PSS-10). Refs. [45,46,47,48,49,50,51] are cite in the Supplementary material file.
