# Supplementary material for: Cardiovascular Health Among Employees of a Brazilian Tertiary Hospital Assessed by the Life’s Essential 8 Score: A Cross-Sectional Pilot Study
Source: J Clin Med. 2026 Apr 20;15(8):3134. doi: 10.3390/jcm15083134 (PMC13116693; doi:10.3390/jcm15083134)
Supplement: Supplementary file 1 [file jcm-15-03134-s001.zip › jcm-4222405-supplementary.pdf]

Health status among employees of a tertiary hospital evaluated by the Life's Essential 8 score

Supplementary Material

Summary

Table S1 –Life`s Essential 8 detailed metrics

Table S2 – Quality of diet - Details of the assessment in the study

Box S1. 10-item Perceived Stress Scale (PSS -10)

Domain	CVH metric	Method of measurement	Quantification of CVH metric in adults
**Health behaviors**	**Diet**	Self-reported daily intake of a DASH-style eating pattern (tool: MEPA for individuals)	Scoeing (individual):<br>MEPA score — LE8 Points<br>15-16 — 100<br>12-14 — 80<br>8-11 — 50<br>4-7 — 25<br>0-3 — 0
	**Physical activity**	Self-reported minutes of ≥ moderate physical activity per week	Minutes — LE8 Points<br>≥150 — 100<br>120-149 — 90<br>90-119 — 80<br>60-89 — 60<br>30-59 — 40<br>1-29 — 20<br>0 — 0
	**Nicotine exposure**	Self-reported use of cigarettes or inhaled NDS or secondhand smoking	Status — LE8 Points<br>Never smoker — 100<br>Fromer smoker (quit ≥5 years) — 75<br>Former smoker (quit 1-<5 years) — 50<br>Former smoker (quit <1 year or currently use inhaled NDS) — 25<br>Current smoker — 0<br>- Subtract 20 points (unless score is 0) for living with active indoor smoker in home
	**Sleep health**	Self-reported average hours of sleep per night	Hours — LE8 Points<br>7-<9 — 100<br>9-<10 — 90

			6-<7	70
			5-<6 or ≥10	40
			4-<5	20
			<4	0
**Domain**	**CVH metric**	**Method of measurement**	**Quantification of CVH metric in adults**	
**Health factors**	**BMI**	Body weight (kilograms) divided by height (meters) squared	BMI (kg/m^2)	LE8 Points
			<25	100
			25-29,9	70
			30-34,9	30
			35-39,9	15
			≥40	0
	**Blood lipids**	Plasma total and HDL cholesterol total, with calculation of non-HDL (mg/dL)	Non-HDL-c	LE8 Points
			<130	100
			130-159	60
			160-189	40
			190-219	20
			≥220	0
			- if drug-treated level, subtract 20 points	
	**Blood glucose**	Fasting blood glucose (FBG; mg/dL) or hemoglobin A1c ($Hb_{A1c;}$ %)	Glycemic profile	LE8 Points
			No DM; FBG<100 (or Hb_{A1c}<5,7)	100
			No DM; FBG 100-125 (or Hb_{A1c} 5,7-6,4) – Pre-DM	60
			DM with Hb_{A1c}<7	40
			DM with Hb_{A1c} 7-7,9	30
			DM with Hb_{A1c} 8-8,9	20
			DM with Hb_{A1c} 9-9,9	10
			DM with Hb_{A1c} ≥10	0
	**Blood pressure**	Appropriately measured systolic and diastolic blood pressures	BP (mmHg)	LE8 Points
			<120/80	100

			120-129/<80	75
			130-139/80-89	50
			140-159/90-99	25
			≥160/100	0
			- if drug-treated level, subtract 20 points	

Table S1 - Life`s Essential 8 detailed metrics - adapted from Lloyd-Jones DM, et al. [1]. CVH, cardiovascular health; BMI, body mass index; BP, blood pressure; DASH, Dietary Approaches to Stop Hypertension; FBG, fasting blood glucose; Hb_{A1c}, hemoglobin A1c; HDL, high-density lipoprotein; MEPA, Mediterranean Eating Pattern for Americans; NDS, nicotine-delivery system

Diet: A score of 100 points for the CVH diet metric should be assigned for the top 95th percentile or a score of 15-16 points on the MEPA (for individuals).
Physical activity: Thresholds are based in part on US Physical Activity Guidelines [2]. For adults, each minute of moderate activity should count as 1 minute and each minute of vigorous activity should count as 2 minutes toward the toral for the week.
Nicotine exposure: It is recommended to subtract 20 points for people exposed to indoor secondhand smoke at home, given its potential for long-term effects on cardiopulmonary health [3].

Sleep health: Thresholds are bases in part on sleep guidelines [4]. Clinicians may consider subtracting 20 points from. The sleep score for people with untreated or undertreated sleep apnea if information is available. Note that overall scoring reflects the inverse-U-shaped association of sleep duration and health outcomes.
BMI: Thresholds are bases in part on National Heart, Lung and Blood Institute (NHLBI) guidelines [5]. It is acknowledged that BMI is an imperfect metric for determining healthy body weight and body composition. Nonetheless, it is widely available and routinely calculated in clinical and research settings. BMI ranges may differ for individuals from diverse ancestries. Clinicians may want to assign 100 points for overweight individuals (BMI, 25.0–29.9 kg/m2) who are lean with higher muscle mass. For underweight individuals (<18.5 kg/m2 in adults or below the fifth percentile in children), the writing group defers to clinician judgment in assigning points on the basis of individual assessment as to whether the underweight BMI is healthy or unhealthy. Conditions that should be considered unhealthy include chronic catabolic illnesses (eg, cancer), eating disorders, and growth failure.
Blood lipids: Thresholds are based in part on 2018 Cholesterol Clinical Practice Guidelines [6]. The levels of non–HDL cholesterol for adults were selected on the basis of current guideline recommendations and in concert with the observation that non–HDL cholesterol levels are generally ≈30 mg/dL higher than low-density lipoprotein cholesterol levels in normative ranges in the population. he writing group recommends subtracting 20 points from the blood lipid score if the level of non–HDL-cholesterol represents a treated value, given the residual risk present in those who require treatment.
Blood glucose: Thresholds are based in part on American Diabetes Association guidelines [7]. If an individual patient with prediabetes (ie, not yet diagnosed formally with diabetes) is being treated with metformin to prevent the onset of diabetes and has normoglycemic levels, the writing group recommends clinician judgment for assigning point values (ie, consider subtracting 20 points). The maximal point value for patients with well-controlled diabetes was set at 40, given the residual risk present in those with diabetes.

Blood pressure: Thresholds are based in part on the 2017 Hypertension Clinical Practice Guideline [8]. It is recommended to subtract 20 points from the BP score if the level of BP represents a treated value, given the residual risk present in those who require treatment

**Component**	**Number of portions for maximum scoring (10 points)**
Fruits	≥ 2 170g-portions/day
Vegetables	≥ 6 30g-portions/day
Fish and seafood	≥ 2 100g-portions/week
Red meat	< 2 100g-portions/semana
Sweetened beverages	< 1 liter/week
Whole cereals	≥ 3 30g-portions/day
Legumes	≥ 1 80g-portion/day
Oilseeds (nuts)	≥ 3 30g-portions/week
Processed meats	≤ 3 30g-portions/week
Dairy products	≥ 1 250g-portion/day
Ultra processed food	< 4 units/day

Table S2 – Quality of diet - Details of the assessment in the study. Number of portions for the Cardiovascular Health Diet Index (CHDI) components considering the maximum score (10 points/component) [9].

Boxes

In the last month, how often						
**1**	Have you been upset because of something that happened unexpectedly?	0	1	2	3	4
**2**	Have you felt that you were unable to control the important things in your life?	0	1	2	3	4
**3**	Have you felt nervous and stressed?	0	1	2	3	4
**4**	Have you felt confident about your ability to handle your personal problems?	0	1	2	3	4
**5**	Have you felt that things were going your way?	0	1	2	3	4
**6**	Have you found that you could not cope with all the things that you had to do?	0	1	2	3	4
**7**	Have you been able to control irritations in your life?	0	1	2	3	4
**8**	Have you felt that you were on top of things?	0	1	2	3	4
**9**	Have you been angered because of things that happened that were outside of your control?	0	1	2	3	4
**10**	have you felt difficulties were piling up so high that you could not overcome them?	0	1	2	3	4

Box S1. 10-item Perceived Stress Scale (PSS -10) . Adapted fom Reis et al.[10]

References

1. Lloyd-Jones DM, Allen NB, Anderson CAM, Black T, Brewer LC, Foraker RE, Grandner MA, Lavretsky H, Perak AM, Sharma G, Rosamond W; American Heart Association. Life's Essential 8: Updating and Enhancing the American Heart Association's Construct of Cardiovascular Health: A Presidential Advisory From the American Heart Association. Circulation. 2022 Aug 2;146(5):e18-e43.
2. Mozaffarian D, Afshin A, Benowitz NL, Bittner V, Daniels SR, Franch HA, Jacobs DR Jr, Kraus WE, Kris-Etherton PM, Krummel DA, et al; on behalf of the American Heart Association Council on Epidemiology and Prevention, Council on Nutrition, Physical Activity and Metabolism, Council on Clinical Cardiology, Council on Cardiovascular Disease in the Young, Council on the Kidney in Cardiovascular Disease, Council on Peripheral Vascular Disease, and the Advocacy Coordinating Committee. Population approaches to improve diet, physical activity, and smoking habits: a scientific statement from the American Heart Association. Circulation. 2012;126:1514–1563.
3. Raghuveer G, White DA, Hayman LL, Woo JG, Villafane J, Celermajer D, Ward KD, de Ferranti SD, Zachariah J; on behalf of the American Heart Association Committee on Atherosclerosis, Hypertension, and Obesity in the Young of the Council on Cardiovascular Disease in the Young; Behavior Change for Improving Health Factors Committee of the Council on Lifestyle and Cardiometabolic Health and Council on Epidemiology and Prevention; and Stroke Council. Cardiovascular consequences of childhood secondhand tobacco smoke exposure: prevailing evidence, burden, and racial and socioeconomic disparities: a scientific statement from the American Heart Association [published correction appears in Circulation. 2016;134:e366]. Circulation. 2016;134:e336–e359.
4. Watson NF, Badr MS, Belenky G, Bliwise DL, Buxton OM, Buysse D, Dinges DF, Gangwisch J, Grandner MA, Kushida C, et al. Recommended amount of sleep for a healthy adult: a joint consensus statement of the American Academy of Sleep Medicine and Sleep Research Society. Sleep. 2015;38:843–844.
5. Clinical Guidelines on the Identification, Evaluation, and Treatment of Overweight and Obesity in Adults. Bethesda, Md: National Heart, Lung, and Blood Institute; 1998.
6. Grundy SM, Stone NJ, Bailey AL, Beam C, Birtcher KK, Blumenthal RS, Braun LT, de Ferranti S, Faiella-Tommasino J, Forman DE, et al. 2018 AHA/ACC/AACVPR/AAPA/ABC/ACPM/ADA/AGS/APhA/ASPC/NLA/PCNA guideline on the management of blood cholesterol: a report of the American College of Cardiology/American Heart Association Task Force on Clinical Practice Guidelines. Circulation. 2019;139:e1082–e1143.
7. American Diabetes Association. Practice Guidelines Resources. Accessed February 15, 2022. https://professional.diabetes.org/content-page/practice-guidelines-resources
8. Whelton PK, Carey RM, Aronow WS, Casey DE Jr, Collins KJ, Dennison Himmelfarb C, DePalma SM, Gidding S, Jamerson KA, Jones DW, et al. 2017 ACC/AHA/AAPA/ABC/ACPM/AGS/APhA/ASH/ASPC/NMA/PCNA guideline for the prevention, detection, evaluation, and management of high blood pressure in adults: a report of the American College of Cardiology/American Heart Association Task Force on Clinical Practice Guidelines [published correction appears in Hypertension. 2018;71:e140–e144]. Hypertension. 2018;71:e13–e115.
9. Cacau LT, et al. The AHA Recommendations for a Healthy Diet and Ultra-Processed Foods: Building a New Diet Quality Index. Front Nutr. 2022;9:804121. doi: 10.3389/fnut.2022.804121.

10. Reis RS, Hino AA, Añez CR. Perceived stress scale: reliability and validity study in Brazil. J Health Psychol. 2010 Jan;15(1):107-14. doi: 10.1177/1359105309346343. PMID: 20064889.
